# Novel aspects in diagnostic approach to respiratory patients: is it the time for a new semiotics?

**DOI:** 10.1186/s40248-017-0098-z

**Published:** 2017-06-27

**Authors:** Gino Soldati, Andrea Smargiassi, Alberto A. Mariani, Riccardo Inchingolo

**Affiliations:** 1 Emergency Department, Valle del Serchio General Hospital, Castelnuovo Garfagnana (Lu), Italy; 2 Pulmonary Medicine Department, University Hospital “A. Gemelli” - Università Cattolica del Sacro Cuore, Largo Gemelli, 8, 00168 Rome, Italy

**Keywords:** Abduction, Chest ultrasonography, Diagnostic approach, Lung, Semiotics, Ultrasound

## Abstract

Medical approach to patients is a fundamental step to get the correct diagnosis. The aim of this paper is to analyze some aspects of the reasoning process inherent in medical diagnosis in our era. Pathologic signs (anamnestic data, symptoms, semiotics, laboratory and strumental findings) represent informative phenomena to be integrated for inferring a diagnosis. Thus, diagnosis begins with “signs” and finishes in a probability of disease. The abductive reasoning process is the generation of a hypothesis to explain one or more observations (signs) in order to decide between alternative explanations searching the best one. This process is iterative during the diagnostic activity while collecting further observations and it could be creative generating new knowledge about what has not been experienced before. In the clinical setting the abductive process is not only theoretical, conversely the physical exploitation of the patient (palpation, percussion, auscultation) is always crucial. Through this manipulative abduction, new and still unexpressed information is discovered and evaluated and physicians are able “to think through doing” to get the correct diagnosis. Abductive inferential path originates with an emotional reaction (discovery of the signs), step by step explanations are formed and it ends with another emotional reaction (diagnosis). Few bedside instruments are allowed to physicians to amplify their ability to search for signs. Stethoscope is an example. Similarities between ultrasound exploration and percussion can be found. Bedside ultrasonography can be considered an external amplifier of signs, a particular kind of percussion and represents a valid example of abductive manipulation. In this searching for signs doctors act like detectives and sometimes the discovering of a strategic, unsuspected sign during abductive manipulation could represent the key point for the correct diagnosis. This condition is called serendipity. Ultrasound is a powerful tool for detecting soft, hidden, unexpected and strategic signs.

## Introduction

Establishing diagnosis is a fundamental step of medical practice: the ability to transform perceptive data into an actionable diagnosis is paramount to the functioning and identity of every physician. Today, however, logical questions for improving diagnosis are largely under-mentioned in medical literature, subservient to evaluating evidence for treatment decisions [1]. Medical school educators devote more time to research on treatment than to diagnosis. Moreover, the progress in diagnosis is commonly associated to the technological development which allows many specialists to inspect part of the human body in a very specific but sectorial way, contributing to the spatial distribution (and distortion) of information. Finally, for at least the last 40 years, evidence based medicine [2] has allowed to understand and apply a hierarchy of evidence, but it is not unequivocally demonstrated that this approach is superior to the experience of the physician, or, in other words, to the so called clinical acumen, especially during the diagnostic bedside activity.

The thesis on which this article is based states that the logic of medical diagnosis, *in primis*, must be studied in terms of judgment under uncertainty [3], a theoretic and manipulative task well known in economics, psychology and sociology, but often forgotten in clinical medicine. This is the necessary step to which every technological evolution must adapt, otherwise the choices will be irrational, therefore unsustainable in practical and especially in economic terms.

On these basis, the aim of this paper is to analyze some aspects of the reasoning process inherent in medical diagnosis. Specifically, a method of clinical bedside diagnosis focused on the manipulative amplification of cardiopulmonary signs by ultrasound, useful for an abductive inferential path [4] dedicated to the pulmonary specialist, will be described and discussed.

## Signs and symptoms

In medicine, signs always appear in conjunction with symptoms and, in general terms, the symptom is felt and the sign is observed. Therefore, the symptom belongs to the “world of introspection of the patient”, as a subjective evidence of disease perceived by the patient, while clinical signs are objective evidence of disease perceptible by the examining health-care provider.

However, the significance of symptom/sign terminology is often imprecise, inconsistent or both, and many medical text, including those dedicated to medical semiotics, fail to provide preliminary definitions of these words. In our diagnostic practice, we privilege the comprehensive term “signs” as the relevant information about patient. This approach represents a Peircean view, where the symptom is only a kind of sign or, in other words, we think that everything that is currently a sign or symptom could instead be represented as an informative clinical finding (the “index sign”) [5].

Pathologic signs, in their broad significance (anamnestic data, symptoms, signs, laboratory and strumental findings), are therefore central in medicine, because they represent informative phenomena to be integrated for inferring a diagnosis. In the process of anamnestic collection, for example, also the communication, disease perception, and relation between doctor and patient are important and could influence the medical report [6].

Clinical judgment, as it was described by Feinstein in a series of classical papers [7–9], is a sequential (often iterative) process which starts from the input data of the patient’s manifestations of disease (index signs) to the output result of diagnostic entities. Thus, diagnosis begins with “signs” and finishes in a probability of disease. The functional space between signs and putative disease is filled with the logic structure of the clinical judgment.

In this way, pathologic signs for the pneumologist are singular information represented by fever, dyspnea, cough, cyanosis, hypoxemia, chest pain, pleural effusion, pulmonary consolidations, interstitial signs, and so on. The evidence for each sign, whatever its perceptive order is, indicates some anatomic or physiological damage and, consequently, it requires the best explanation according to an abductive process [10–12].

## The abductive process

Abduction is basic for both diagnostic and experimental hypothesis, it is common in scientific discovery and in daily bedside clinical judgment, formerly analogous to a bedside “experiment”.

In simple terms, abduction is the generation of a hypothesis to explain one or more observations. While explaining a given set of observations (signs), the clinician has often to decide between alternative explanations to search the best explanation (in probabilistic terms) according to the observations [12, 13]. During the diagnostic activity, as in many real world tasks, abduction is an iterative process, because succeeding observations are sequentially interpreted and integrated to generate a single current explanation for all captured signs. The current explanation is never absolute, but it is modulated in its significance by the flow of the observations. On the other hand, every new observation has a corroborative (or adverse) value with respect to the current explanation [14, 15]. Thus the current explanation acts as an explanatory context for the comprehension and explanation of new observations, unless new observations are characterized by a high degree of specificity to require a better explanation. This dynamic process ends when the flow of observations is stopped because, for example, the explanatory power of the whole process is deemed sufficient (secure inferential reasoning).

So, abduction has a logical form, distinct from deduction (and induction), because, contrary to deduction, it starts from consequences and looks for reasons (retroduction), and because it represents a creative process, generating new knowledge. In other words, reasoning involved in abduction amplifies, or goes beyond, the information incorporated in the premises. According to Magnani [13, 16], abduction can generate “plausible” hypotheses (“creative” abduction), or it can be simply considered as inference “to the best explanation”, which evaluates prestored hypotheses. For example, the discovery of a new disease and the manifestations it causes, can be considered as the result of a creative abductive inference (similarly to a scientific discovery), while in usual medical diagnosis, the task of the expert physician is to “select” the best explanation from an encyclopedia of prestored diagnostic entities.

Therefore, though every clinical judgment is abductive, not all abductive processes are equal [17]. In hyper-codified abduction an inferential phase exists, got through every time any given sign appears. In pneumological practice, massive pleural effusion implies dyspnea, and, consequently, a thoracic dullness, (pleural effusion) may be the best explanation for a dyspnea. However, in other circumstances the inferential role must be selected from a number of equally pertinent options already known to the physician. For example, thoracic auscultatory crackles may be ascribed to either edema or fibrosis, and a similar question arises when the clinician discovers an interstitial sonographic syndrome [18]. This kind of abduction, in which an univocal relationship between sign and explanation does not exist, is called hypo-codified abduction [17]. Obviously, it requires some form of iteration, or an external instrument, to achieve an acceptable statistical significance. Finally, in creative abduction [19], the rules must be found ex novo. In this case, the inquirer is confronted with puzzling facts, but there is no knowledge of a law or a general role that may explain the facts. He must conceive the explanation itself. Despite creative abduction deals with the whole field of the growth of scientific knowledge, its educational role in clinical medicine should not be underestimated. In our opinion, a subset of creative abduction is a powerful tool also in the usual clinical medicine: for the clinician with limited experience or in training, when he or she needs to hypothesize or explain what has not been experienced before, and for physicians with extensive knowledge upon finding a relatively new, strange, unusual situation [20].

## Thinking through doing

According to our idea, in Peircean sense, diagnostic inferences are a form of sign activity, where the word “sign” includes feeling, image, conception, and other representations (in clinical terms: anamnestic data, symptoms, signs, laboratory and instrumental findings). Abduction is the process of reasoning, often necessarily iterative (non-monotonic character of abductive reasoning), in which explanatory hypotheses, based on sign activity, are dynamically formed and evaluated (abductive space).

Another aspect deserves attention. In the clinical setting the abductive process is not only argumentative or theoretical, but always the physical exploitation of the patient is crucial. Through this environmental handling (manipulation) new and still unexpressed information are discovered and evaluated. Therefore, manipulative abduction is a necessary adjunct to theoretical abduction and it happens when we are thinking through doing and not only, in a pragmatic sense, about doing [16, 21]. In any way the logic of diagnosis is carried out, this old but still valid concept guides the clinical activity and even the traditional semiotics obviously uses manipulative actions (palpation, percussion, auscultation) for eliciting signs.

During the manipulative abduction the clinician captures many interesting signs through actions that can provide otherwise unavailable information, so he or she is able to solve problems by starting and performing a suitable abductive process of generation or selection of hypotheses.

These actions, performed in order to receive particular kinds of sensorial stimulation that contain information otherwise unavailable, are strictly related to the argumentative or theoretically arranged perceptions: therefore, perception can cause action, but also action can cause and control perception [22]. For example: a dyspnoic patient is observed, the sign dyspnea is only an inspective information and the theoretical explanations for this sign may be edema, pleural effusion, pneumonia, pneumothorax, and so on. These explanations represent a too large set of hypothesis for a correct diagnosis. However, a best explanation can be formulated when the doctor uses his hands, or the stethoscope, on the thorax of the patient to detect sounds, vibrations or resonances, in this way structuring an additional, perceptive action (manipulation).

## External amplifiers

Many instruments are used for examining the patient, but few are available to every physician and usable bedside. Stethoscope acts as sensorial mediator, that is, a simple instrument that give the doctor the possibility to perceive data in a more practical and sensitive way. Except for this instrument, the doctor has few other tools for its diagnostic activity addressed by traditional semiotics. Usually, as in the case of palpation and percussion, he or she structures basic natural manipulative actions producing raw perceptions (visual, tactile and auditory).

Recently the use of ultrasound has spread to many diagnostic settings. During the last years, many Respiratory Medicine specialists have acquired the necessary skills to define pathological conditions of the lung and pleura at the bedside and in respiratory intensive unit [23, 24]. The presence of an ultrasound machine is also strongly recommended in the pleural disease room and for guiding interventional procedures. This instrument exploits the properties of acoustic waves in order to acquire structural and functional information about internal tissues and organs otherwise non-explored with the usual clinical maneuvers. It represents a rare case in which a natural, non-detrimental energy (the sound) and its properties are used as a tool to explore the body internal tissues and organs, so that this manipulation can give rise to some relevant knowledge [25]. Thus, the similarities between ultrasound exploration and percussion are intriguing. This is the reason why we consider bedside ultrasound not as instrumental diagnostic method, but as an external amplifier of signs, that is, a particular kind of percussion. Therefore, the ultrasound machine in pulmonology is nothing more than an external amplifier and the sonographic examination represents a valid example of abductive manipulation.

## Clinical example

**Fig. 1:**
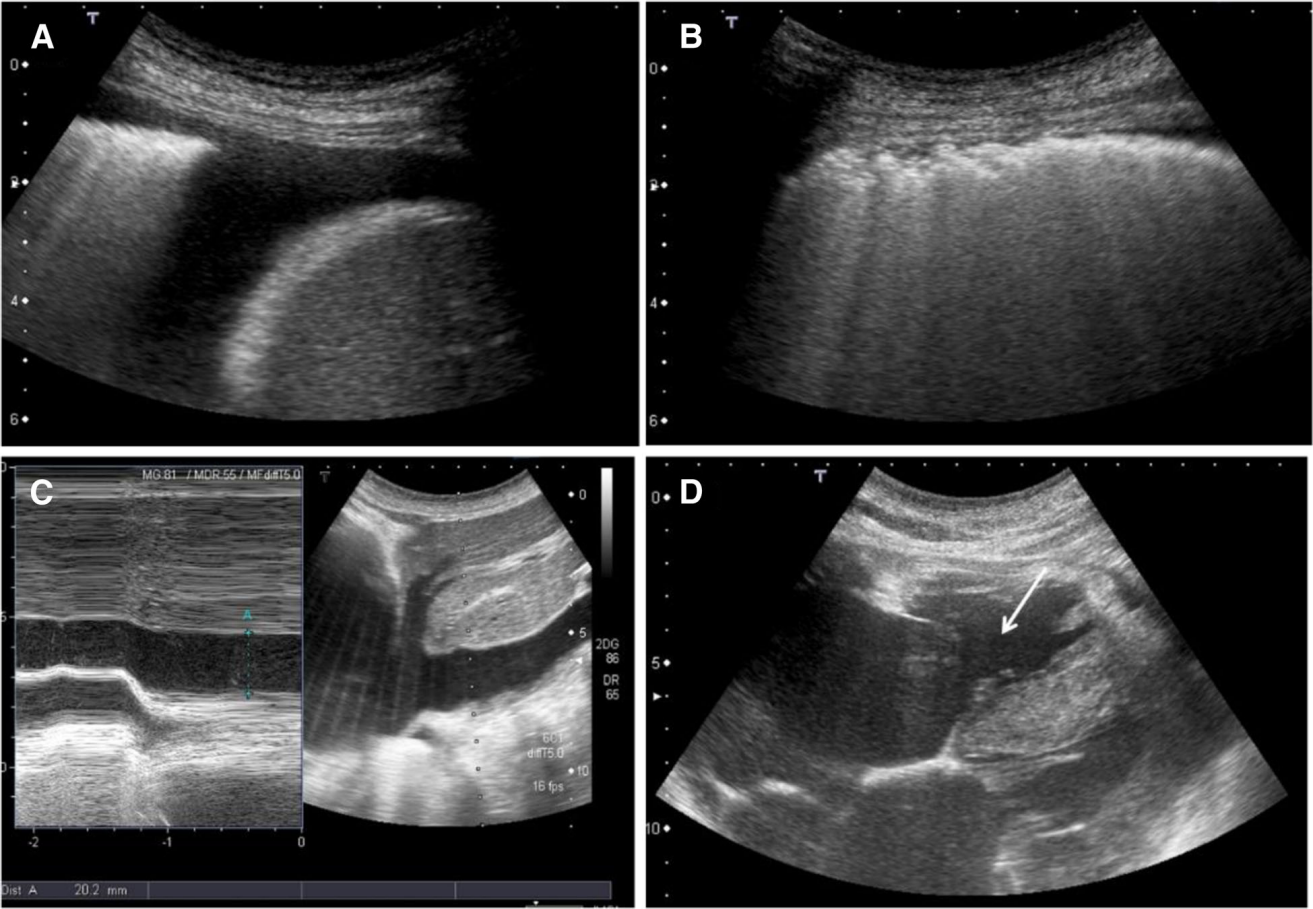
Seventy-five old man with persistent cough, shortness of breath of recent onset and ankle edema. **a**: Small transonic pleural effusion on the right side. **b**: Pneumogenic Interstitial Syndrome. The pleural line is irregular and the sub-pleural plane is white, without horizontal reverberations and mirror effect. **c**: Inferior cava vein is enlarged without inspiratory collapse. **d**: Sub-xiphoidal view of the heart showing enlarged right ventricle (*arrow*) with thickened wall. The left ventricle is normally kinetic

**Fig. 2:**
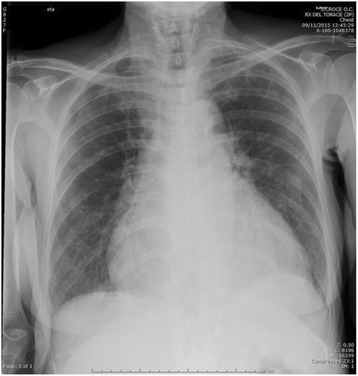
Chest x-ray of the same patient showing enlarged cardiac shadow and uncertain signs of pleural effusion and interstitial lung disease

This case (Figs. 1 and 2) is deliberately described through scarce and poor documented findings, in order to make the abductive approach rather elementary.

A 75-old man consults the physician for persistent cough, shortness of breath of recent onset and ankle edema. He is alert anxious and tachypneic, his blood pressure is normal, his oxygen saturation is 94% while receiving oxygen via nasal cannula at 2Lpm. His lung exam has diminished breath sounds bilaterally and no wheezes or rhonchi are noted. Rare crackles are heard over the lung bases. Cardiac exam shows an holosystolic murmur on the centrum.

- Step 1: Immediate (hyper-codified) theoretical abduction (on inspective basis) results in a cardiopulmonary problem (the immediate provisional best explanation).
- Step 2: Manipulative abduction (model-based by sensorial information: palpation, percussion and auscultation) results in heart failure (explanation 1) or pulmonary parenchymal disease (explanation 2). However, we need to choose the best explanation and a too large statistical uncertainty exists.
- Step 3: Manipulative abduction using an external mediator or amplifier. The doctor uses ultrasound (Fig. 1) and the final best explanation is pulmonary fibrosis with chronic cor-pulmonale.

## Stages of abduction

**Fig. 3:**
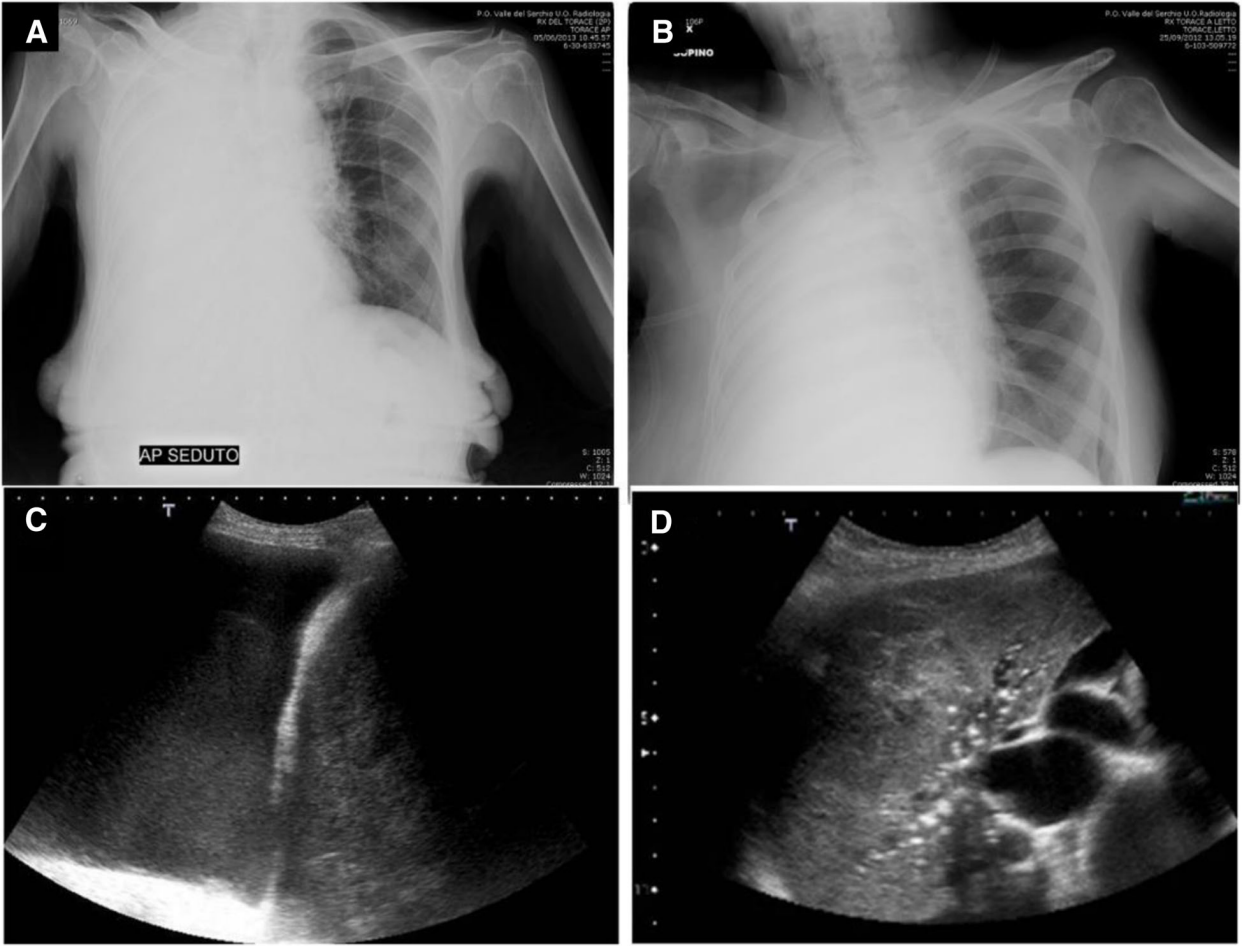
**a** and **b**: Chest x rays showing two patients with opaque right hemithorax. **c** and **d**: ultrasound scans of the right hemithorax in the same patients. The diagnosis is immediate (hyper-codified abduction). **a**. massive pleural effusion. **b**: right lung atelectasis. The right main bronchus is closed by a large hilar mass

Theoretical and manipulative abduction shows a non-monotonic character, because the most likely explanations are not necessarily correct and multiple explanations might exist. Abduction can be represented as a dynamic iterative process where further observations are searched to discriminate among candidate explanations. For example, searching the elementary explanation for a dyspnoic patient with a percussive dullness over an hemithorax is a simple task (hemithorax without air - effusion or atelectasis). However, the exact (best) explanation is reached when an ultrasound scanning immediately shows fluid in pleural space (effusion) or a consolidated lung (atelectasis) (Fig. 3). This case represents a (at least) two step manipulative abduction. That is, we assume that one or more initial observation is given and that there is a way to generate candidate explanations based on them. We then update the candidate explanations based on additional observations. In this sort of dynamics, we proceed with selecting and performing one observation at a time, and, in this setting, the use of an external amplifier (ultrasound) is determining, allowing to perceive data that are not perceivable by means of the natural sensorial ability.

**Fig. 4:**
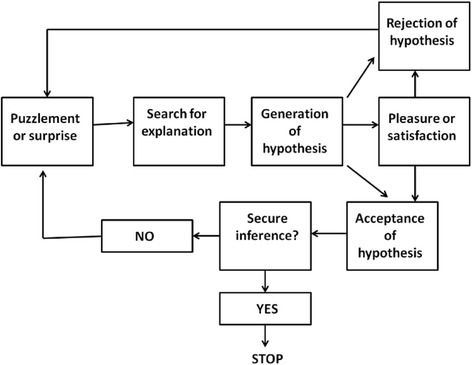
Stages of abduction and its iterative dynamics, searching for the best explanation (27, modified)

Abduction is a multi-staged mental and model based process [22]. The diagnostic process begins with the evidence that something puzzling (a pathologic “sign”), that prompts the physician to generate an explanation, exists. Consequently, the mind searches for possible hypotheses that could explain the “sign”. Sometimes the search is easily completed when there is a prestored hypothesis waiting to be applied (hyper-codified abduction). Other times the search for an explanation is more complex [17]. In every case, this process generates a candidate explanatory hypothesis to be assessed with respect to competing hypotheses (inference to the best explanation). Finally, the candidate hypothesis can be accepted, revised or rejected. Except when the hypothesis is finally accepted, this iterative process remains active for searching the best explanation [26] (Fig. 4).

## When puzzling is serendipitous

Just as abduction originates with an emotional reaction (the discovery of the sign), it ends with another emotional reaction (satisfaction and pleasure to get diagnosis) [27]. These emotional reactions are greater when the explanatory hypothesis is plausible, such it can be considered definitive (the correct diagnosis). Similarly physicians experience great astonishment and surprise whenever a strategic, unsuspected, hidden, explanatory sign is discovered.

Ultrasound is a powerful tool for detecting soft, hidden, unexpected and strategic signs. For this reason, we consider ultrasound an external epistemic mediator and amplifier that should be employed by every doctor. According to this view, ultrasonography is nothing more than a powerful semiotic maneuver.

Ultrasound is sensitive regarding many pathologies, frequently it detects definitive signs of disease and, not least, it allows to discover strategic and unsuspected signs able to change the diagnostic pathway [28–33].

**Fig. 5:**
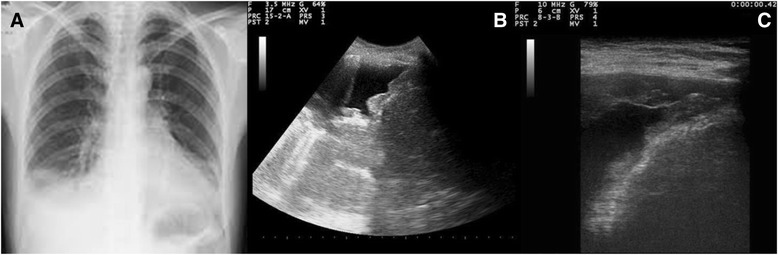
**a**: patient with right pleural effusion, plain chest x-ray. **b** and **c**: Bedside Chest ultrasonography strategically revealing nodular variations of the parietal pleura, diaphragm and costophrenic sinus. This serendipitous finding immediately orients towards the most likely diagnosis (neoplastic pleural involvement)

For example, we are able to detect and describe pleural effusions with classic semiotics and Chest X-ray. But chest ultrasonography, performed bedside, can strategically reveal small details and abnormalities that immediately orient towards the most likely diagnosis. In the case of Fig. 5, evidence of nodular variations of the parietal pleura, diaphragm and costophrenic sinus in the ultrasonographic approach to pleural effusion, immediately orients towards neoplastic pleural involvement (Fig. 5)

This condition is called serendipity. The term refers to the fairy tale “The three Princeps of Serendip” (an old name of Sri Lanka) who were able to perfectly describe a camel from small incidental details using sagacity and without having never seen it before [34].

The New Oxford Dictionary of English defines serendipity as the occurrence and development of events by chance in a satisfactory or beneficial way, understanding the chance as any event that takes place in the absence of any obvious project (randomly or accidentally).

Serendipity and ultrasound are strictly linked, they can play an important role in the search for truth, but this view is often ignored in scientific literature because traditional medical behavior and scientific thinking is based on logic and predictability [35].

## Clues, doctors and detectives

Clues (signs) represent a link between some thought processes and drives necessary for both medical and detective work. In the light of previous discussion, the physician must acquire knowledge and an abductive logic similar to the detective’s art. In 1970 Carlo Ginzburg published the first version of his essay “Spie: Radici di un paradigma indiziario” [36]. The aim of this article was to direct attention to the breakthrough of a new epistemological model in human sciences, that was called “the evidentiary paradigm” or “the method of clues”. This method means interpreting insignificant and marginal information as clues. Ginzburg compared common features in the method used by the art historian Giovanni Morelli, Sherlock Holmes and Sigmund Freud, suggesting potential common contacts among these thinkers. In all three cases, tiny details (symptoms for Freud, clues for Holmes and features of painting for Morelli) provide the key to a deeper reality, inaccessible by other methods [36]. It is certainly fascinating to discover that Freud was a doctor, Morelli had a degree in Medicine and Conan Doyle had been a doctor before settling down to write. In all three cases the model of medical semiotics appears.

Nordby [37] has exemplified the centrality of abduction to the work of forensic scientists and Innes [38] to that of detectives, concluding that abduction is far the most commonly deployed form of investigative logic employed on murder enquiries.

According to these considerations, it can be argued that a richer understanding of abduction (in the form of evidentiary paradigm) in many settings, including the medical diagnosis, could enhance the quality of investigation to generate more and better hypothesis, make them more sensitive to potential errors in perception, comprehension and construction, and inhibit improper convictions [39].

## Conclusions

It is the opinion of the Authors that the clinical diagnosis in many specialties, including Pulmonology, should re-evaluate and enhance the meaning of the signs in a new semiotic view. Pathologic signs (in their broad sense: anamnestic data, symptoms, signs, laboratory and strumental findings) represent information to be channeled and analyzed in a abductive inferential path, where, step by step, explanations are formed. This dynamic, iterative process has the purpose of perfecting the best final explanation (“the secure explanation”, in probabilistic terms). In other words, the doctor chooses perceptive actions that allow him to receive a feeling or a structure of feelings which clearly and easily provides the diagnostician with specific information to be explained. Anyway, besides the use of bodily actions, external instruments must be used for personally acquiring additional data, and this step is strongly recommended.

The great, recent, development of thoracic ultrasound in the hands of the respiratory physician suggests that this non-invasive technology can be the ideal candidate to become an extraordinary external epistemic mediator. Its sensitivity for discovering mild signs, marginal but strategic information, and clues, allows a modern and intelligent re-evaluation of serendipity as a valuable tool for a better knowledge, according to an evidentiary paradigm.
